# Temporal organization of rest defined by actigraphy data in healthy and childhood chronic fatigue syndrome children

**DOI:** 10.1186/1471-244X-13-281

**Published:** 2013-11-04

**Authors:** Minako Kawabata, Taro Ueno, Jun Tomita, Junko Kawatani, Akemi Tomoda, Shoen Kume, Kazuhiko Kume

**Affiliations:** 1 Department of Stem Cell Biology, Institute of Embryology and Genetics, Kumamoto University, Kumamoto, Japan; 2 Kuwamizu Hospital, Kumamoto, Japan; 3 Department of Child Development, Faculty of Life Sciences, Kumamoto University, Kumamoto, Japan; 4 Research Center for Child Mental Development, Fukui University, Fukui, Japan; 5 Department of Neuropharmacology, Graduate School of Pharmaceutical Sciences, Nagoya City University, Tanabe Street, Mizuho, Nagoya 467-8603, Japan

**Keywords:** Sleep, Temporal organization, Activity, Rest, Actigraphy

## Abstract

**Background:**

Accumulating evidence has shown a universality in the temporal organization of activity and rest among animals ranging from mammals to insects. Previous reports in both humans and mice showed that rest bout durations followed long-tailed (i.e., power-law) distributions, whereas activity bouts followed exponential distributions. We confirmed similar results in the fruit fly, *Drosophila melanogaster*. Conversely, another report showed that the awakening bout durations, which were defined by polysomnography in bed, followed power-law distributions, while sleeping periods, which may correspond to rest, followed exponential distributions. This apparent discrepancy has been left to be resolved.

**Methods:**

Actigraphy data from healthy and disordered children were analyzed separately for two periods: time out of bed (UP period) and time in bed (DOWN period).

**Results:**

When data over a period of 24 h were analyzed as a whole, rest bouts showed a power law distribution as previously reported. However, when UP and DOWN period data were analyzed separately, neither showed power law properties. Using a newly developed strict method, only 30% of individuals satisfied the power law criteria, even when the 24 h data were analyzed. The human results were in contrast to the *Drosophila* results, which revealed clear power-law distributions for both day time and night time rest through the use of a strict method. In addition, we analyzed the actigraphy data from patients with childhood type chronic fatigue syndrome (CCFS), and found that they showed differences from healthy controls when their UP and DOWN data were analyzed separately.

**Conclusions:**

These results suggested that the DOWN sleep, the bout distribution of which showed exponential properties, contributes to the production of long-tail distributions in human rest periods. We propose that separate analysis of UP and DOWN period data is important for understanding the temporal organization of activity.

## Background

Most natural phenomena appear to occur stochastically and this random distribution of phenomena is thought to produce Poisson distributions. However, it has been shown recently that many phenomena, such as human behavior, form non-Poisson distributions. For example, interevent intervals of social behaviors such as e-mail communications and trade transactions follow power-law distributions [1]. Nakamura et al. showed that rest bout durations followed long-tailed (i.e., power-law) distributions, whereas activity bouts followed exponential distributions in both humans and mice [2,3]. A similar temporal organization of rest and activity bouts has also been observed in invertebrates. In the insect *Drosophila melanogaster*, waiting intervals between behavioral episodes such as walking, feeding, and flight maneuvers follow the power-law distribution [4-8]. Using a video-recording method, we recently confirmed that the duration of the rest bout for the flies followed a power-law distribution [9], even when a strict method described by Clauset et al. [10] was employed. We recently clarified the dopaminergic circuit regulating arousal, which will bring insight how these temporal organization is installed in the brain circuit [11].

In contrast, the use of an electroencephalogram during bed time has shown that awakening bout durations follow power-law distributions, while sleeping periods follow exponential distributions [12]. This discrepancy is unexpected, since wakefulness and sleep are thought to correspond to activity and rest, respectively. We wanted to solve this apparent discrepancy, since actigraphy is more handy and versatile than polysomngraphy in routine situations and its importance in clinical diagnosis is increasing [13-16]. Therefore, in order to address this issue, we analyzed human actigraphy data that was separated into time out of bed (UP) and time in bed (DOWN) periods. We also examined actigraphy data from childhood chronic fatigue syndrome (CCFS) patients. Our results revealed that separate analysis of UP and DOWN period data is crucial for understanding the temporal organization of activity, and that the rest bout during DOWN period, most of which correspond to sleep, shows exponential distributions, and this plays an important role for the production of long-tail distributions in human rest periods.

## Methods

### Subjects and ethical consideration

Healthy control and CCFS patient subjects were recruited as described previously [17]. Briefly, CCFS patients were recruited from patients who visited Kumamoto University Hospital between April 2007 and December 2008 because of CFS-like symptoms. 127 patients with CCFS diagnosis were assessed and 70 (37 male, 33 female, age 9 to 18 y. o.) of them were enrolled. Healthy control subjects were recruited from the regional middle school, and 34 healthy middle school teenager subjects (15 male, 18 female, 1 unknown sex, 13 to 15), underwent actigraphy examination. This study was approved by the institutional review board of Kumamoto University. Written consents were obtained from all of the subjects.

### Actigraphy

Actigraphy examinations were performed using a Micro-Mini Motionlogger (Ambulatory Monitoring Inc., AMI, NY, USA) with Zero-Cross mode (ZCM) using 1 min bins, which was worn on the wrist of the non-dominant arm. In ZCM mode, the actigraph counts the number of times the accelerometer waveform crosses 0, which means the gravity velocity value changes either from minus to plus or from plus to minus, for each time period. Subjects were asked to wear it at all times over a two week period, except for when they bathed or engaged in hard physical exercise.

After collection of the data, we manually screened all the data and excluded subjects who met the following criteria: (1) when the total recorded period was less than 3 days; (2) when more than half of the data was bad; or (3) when the data was clearly abnormal, which suggested a problem with the instrument. As a result of this screening process, we used 23 healthy control subjects (10 male, 12 female, 1 unknown) and 59 CCFS subjects (30 male, 29 female).

### Data analysis

**Figure 1:**
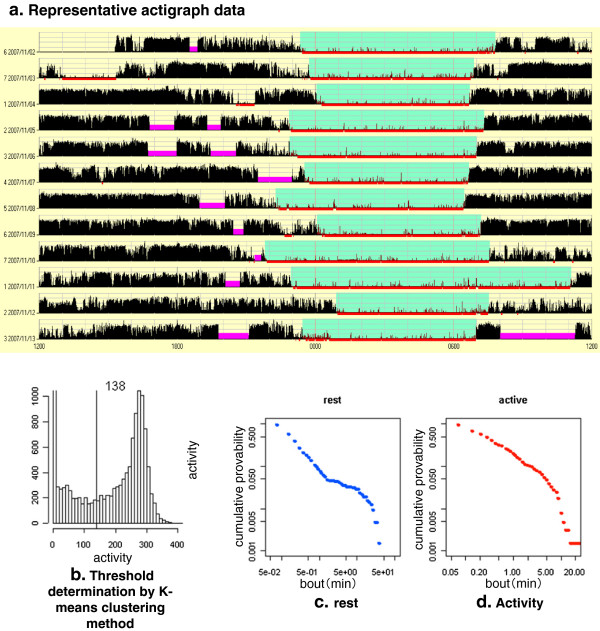
**Actigraphy data analysis. a**, actual actigraphy data from a representative control child. Horizontal row shows activity data over 24 h (12 noon to 12 noon). Black bar indicates the movements in one min, purple lines indicate the bad periods (periods when the subject apparently removed the actigraphy instrument), red underlines indicate the sleep period as calculated by the software using the Kripke-Cole algorithm, and the light blue indicates DOWN periods, when the subjects are thought to be in bed. UP periods are defined as the remaining period after DOWN periods are removed. **b**, histogram of the bins according to the degree of movement. Horizontal bar indicates the number of movement counts in a one minute bin by the actigraphy. The vertical line at 138 indicates the threshold determined by the *k*-means clustering algorithm. **c**, double logarithmic plots of the cumulative probability of the rest bout for this subject. **d**, double logarithmic plots of the cumulative probability of the activity bout for this subject.

Using Action W-2 software from AMI, we manually labeled the bad bins (when the subjects took off the instrument; Figure 1a, colored in purple). The software calculated sleep time using the algorithm by Cole et al. [18]. Then we determined DOWN bins, which denoted the time that the subjects were supposed to be in bed, semi-automatically using the same software and followed this with manual corrections (Figure 1a, colored in light blue). Intervals not labeled as DOWN are labeled as UP. Note that only one continuous DOWN bin interval is labeled per day, and the UP / DOWN intervals basically correspond to UP and DOWN. The raw data (activity counts by ZCM mode, indicator of good/bad, indicator of up/down) for each min bin were then exported to a comma-separated value file.

Then, using custom-made software developed using the mathematical platform R (R-Development-Core-Team), the data was processed. The process and the software are essentially the same as described previously [9]. First, all of the bad bins were removed. ‘Bad bin’ is the period when we assumed the subject removed the actigraph from their wrist, so there was no movements for an extended time. Bad bins are typically observed when the subjects take a bath. They are asked to remove actigraph when they take a bath in a traditional Japanese way, where they soak themselves in a bathtub for an extended time. Second, the threshold value for the activity counts was calculated, using either the averaging method described in Nakamura et al. [3] or the k-means clustering method used in Ueno et al. [9]; Figure 1b, see Results). Third, three classes of data set were made, namely a 24-h data set containing all of the data, a UP data set containing only the UP interval data, and a DOWN data set containing only the DOWN interval data. Fourth, each 1-min bin was classified into one of two categories, namely rest or activity according to the activity threshold value, or a rest or activity episode that was defined by a series of consecutive rest or activity bins, respectively. Thus, rest episodes and activity episodes alternated. The length of the rest or activity episode was defined as a rest or activity bout, respectively. Fifth, the resulting rest and activity bouts were statistically analyzed as described previously [9].

To quantify the distribution of bouts, we calculated the complementary cumulative probability distribution of bouts, *P*(*x≥a*), where *x* is the variable representing the bout, and *a* is the designated duration length. *P*(*x≥a*) represents the fraction of the rest or activity episode that has a length larger than *a* (min), respectively. Using the probability density function *p*(*x*), the cumulative probability is $P\left(x\geq a\right)=\int_{a}^{\infty }p\left(x\right)\mathrm{dx}$. Our main interest is the property of *p*(*x*) and hence *P*(*x*). Cumulative distributions of rest and activity bouts were calculated from the data from individuals (Figure 1c, d).

To test the plausibility of the power-law hypothesis compared to alternative distributions without long tails, we performed a likelihood ratio test [10]. The likelihood ratio is defined as the likelihood of the data under the estimated power law to that under an alternative distribution estimated by the maximum likelihood method. If this value is a large positive number, the power-law assumption is considered to be plausible compared to the alternative distribution. As alternative distributions, we chose a distribution without long tails, i.e., the exponential distribution *p*(*x*) = *λe*^− *λx*^.

Furthermore, we fitted a power-law *P*(*x*) ∝ *x*^− *α* + 1^ to the cumulative distribution of rest or activity bouts using a maximum likelihood method that carefully estimates the lower bound of *x*, as well as *a*[10]. Assuming that our data are drawn from a distribution that follows a power-law, we can derive maximum likelihood estimators. The data are most likely to have been generated by the model using a parameter that maximizes this function. Next, we performed a goodness-of-fit test to determine the plausibility of the power-law hypothesis. To this end, we generated 1,000 surrogate data sets using a nonparametric bootstrap method, each being a set of artificial bouts sampled from the estimated power-law distribution. To calculate the deviation of a distribution, either empirical or artificial, from the estimated power-law distribution, we used the Kolmogorov-Smirnov (KS) statistic, which is the maximum distance between the cumulative distributions of the two distributions to be compared. The resulting *p*-value was defined as the fraction of surrogate realizations, such that the KS distance between the distribution generated from a surrogate data set and the estimated power law is larger than the KS distance between the empirical distribution and the estimated power law. Therefore, a large *p*-value implies that the power-law distribution reasonably fits the original data. Note that the notion of the *p*-value introduced here is different from the standard one, in which a smaller *p*-value in the goodness-of-fit test indicates more significance. To avoid confusion, we refer to the notion of *p*-value introduced here as *p′-*value.

### Ethical considerations

This study was approved by the ethical committee of Kumamoto University. Written informed consents were obtained from the subject or their parent before the study.

## Results

### Threshold validation

**Figure 2:**
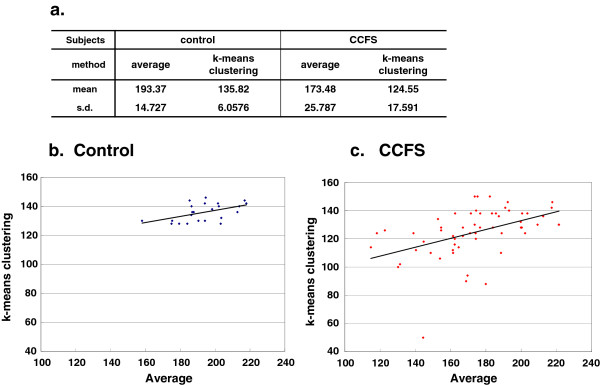
**Threshold value validation. a.** Mean and standard deviation (s.d.) of the threshold values calculated by two different methods (average method and k-means clustering method) of two subject groups (control and CCFS). **b-c.** Correlation between threshold values of two subject groups calculated by two different methods.

First, we investigated whether the difference in the threshold value affected the temporal distribution profile. Nakamura et al. used the modified average value as the threshold. They removed the bins with the value of zero from the calculation of the average and confirmed that the results were stable after changing the threshold value within the range of 0.6 fold to 1.6 fold of the modified average value [2]. As shown in Figure 1b, the activity counts show a clear bimodal distribution. Since we were trying to analyze the temporal distribution of two different states, rest and activity, a clustering method was considered to be more appropriate than an average value. In the previous study, we actually used a k-means clustering method which worked well [9]. We calculated the threshold value using both methods in control and CCFS subjects. As shown in Figure 2a, the modified average method yielded larger values with larger standard deviations than the k-means clustering method. There were, however, significant correlations between the two values (correlation coefficient = 0.21 and 0.31 for control and CCFS subjects). We used k-means clustering in the following analysis since the threshold values calculated were within the range confirmed to give the similar results in the previous study described above.

### Temporal organization of rest and activity of control and CCFS subjects

**Figure 3:**
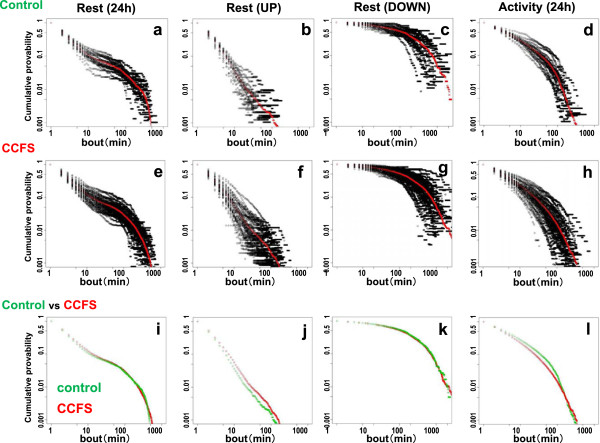
**Double logarithmic plots of the cumulative probability of the rest and activity bouts. a-h**, all of the data from individuals are plotted in black with the average value in red. **i-l**, the average graphs of control (green) and CCFS (red) are plotted. **a-d**, control subjects; **e-h**, CCFS subjects. **a,e,i** represent 24 h rest; **b, f, j**, rest in UP periods; **c, g, k**, rest in DOWN periods; **d, h, l**, activity in 24 h periods.

First, we analyzed and compared the 24 h data from healthy control subjects with CCFS subjects. For both groups, rest durations showed a gradual downslope curve with a slight upward flexion around 10 min, followed by a rapid downward flexion around 100 min (Figure 3a, e). The average curves for the two groups were similar in shape (Figure 3i) and activity durations showed a smooth, continuously decreasing curve for both groups (Figure 3d, h). The shape of the average graphs looked similar, but the CCFS group decreased more rapidly than the control group (Figure 3l).

We then re-analyzed the rest data after separation into UP and DOWN periods. The rest durations during the UP period showed a rapid linear decrease for both groups (Figure 3b, f). The average slopes for the two groups were similar, though the control group revealed a slightly steeper decrease (Figure 3j). The rest durations during the DOWN period showed a smooth, continuously decreasing curve for both groups (Figure 3c, g) and the average curves for the two groups almost completely overlapped (Figure 3k). The shapes for the average curves for rest are remarkably distinct for the 24 h, UP and DOWN periods.

Since almost all the activity episodes during the DOWN periods are short (less than 5 min), there were not enough data of activity during DOWN period for statistical analysis for regression, and the probability distribution of activity during UP period is almost indistinguishable from that during 24H period. In addition, since the main purpose of this study is to examine the power-law properties of rest period distributions, we did not include separately analyzed data of the activity.

### Quantitative analysis of rest duration

**Figure 4:**
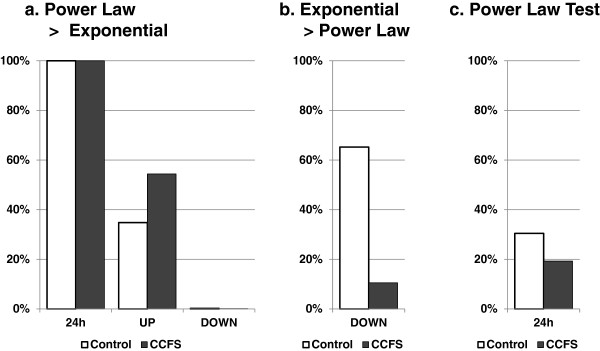
**Power law distribution of rest bouts. a**, comparison between power law distribution and exponential distribution. Values indicate the percentage of individual data that show greater similarity to the power law distribution than to the exponential distribution. **b**, same as a, except that values indicate the percentage of individual data showing greater similarity to the exponential distribution than to the power law distribution. **c**, percentage of individual data judged to be significantly well fitted to the power law.

Next, we quantitatively analyzed the distribution pattern for rest duration. All the results are summarized in Tables 1 and 2. Tables 1 and 2 show the individual data for control and CCFS subjects, respectively. First, we compared the distribution of each subject with power law and exponential distributions using a likelihood ratio test. When the likelihood ratio (LR) was a positive value with a p value less than 0.05, the distribution was significantly closer to the power law than to the exponential. When the LR was a negative value with a p value less than 0.05, it was significantly closer to the exponential than to the power law. Otherwise, there was no significant deviation from either distribution, and thus the distribution favors to neither of them. When the p value is less than 0.05, it is written in red. As summarized in Figure 4a, all cases from both control and CCFS subject groups showed distributions closer to the power law distribution than to the exponential distribution for the 24 h data. However, when the UP period data were analyzed separately, only about 35% of control and 56% of CCFS subjects showed significant similarity to the power law over the exponential distribution. The rest (65% of control and 44% of CCFS) favored neither to the power law nor to the exponential distribution. Moreover, no DOWN data showed any similarity to the power law in either subject group. On the contrary, more than 60% of control and 10% of CCFS DOWN data were similar to the exponential distribution, while the rest (40% of control and 90% of CCFS) favored neither to the power law nor to the exponential distribution (Figure 4b). In addition, we analyzed the 24 h activity data for both groups, but none was judged to be close to the power law (data not shown).

**Table 1:** Statistical tests for the significance of the power-law property in distributions of rest bouts according to the method described by Clauset et al. (control)

**No.**	**Basic parameters**				**Power law vs. exponential**						**Power law test**				
					**24h**		**UP**		**DOWN**						
	**n**	**<x>**	**Total**	**sd**	**LR**	**p**	**LR**	**p**	**LR**	**p**	**xmax**	**xmin**	**α**	**ntail**	**p’**
1	570	12.3	6998	34.8	12.4	*0.00*	1.5	0.13	-6.8	*0.00*	416	1	1.66	570	*0.14*
2	652	10.9	7111	48.0	9.9	*0.00*	1.9	*0.06*	-3.1	*0.00*	468	1	1.82	652	0.01
3	340	18.9	6424	62.8	12.4	*0.00*	1.6	0.12	-0.8	0.40	439	1	1.67	340	0.10
4	627	12.6	7870	53.5	11.7	*0.00*	0.6	0.52	-3.4	*0.00*	660	2	1.87	342	0.00
5	636	10.5	6657	36.0	14.3	*0.00*	1.0	0.31	-2.8	*0.01*	342	1	1.82	636	0.00
6	864	9.3	8005	34.2	11.4	*0.00*	1.3	0.20	-5.1	*0.00*	395	2	1.90	486	0.01
7	782	8.7	6825	35.7	9.1	*0.00*	1.0	0.31	-2.5	*0.01*	420	1	1.80	782	0.00
8	719	10.4	7469	40.7	9.5	*0.00*	1.2	0.23	-2.1	*0.03*	446	2	1.85	407	*0.23*
9	1035	7.8	8083	25.6	12.2	*0.00*	0.9	0.35	-10.6	*0.00*	250	2	1.88	539	0.03
10	687	10.8	7420	47.1	9.7	*0.00*	2.5	*0.01*	-2.6	*0.01*	444	1	1.80	687	0.02
11	561	10.8	6050	34.6	13.7	*0.00*	1.6	0.11	-1.5	0.15	406	1	1.79	561	0.00
12	990	8.9	8828	40.4	8.1	*0.00*	1.0	0.34	-5.3	*0.00*	688	2	1.91	556	0.02
13	759	9.0	6804	20.0	8.3	*0.00*	2.2	*0.03*	-9.8	*0.00*	185	2	1.73	449	0.00
14	680	11.4	7738	38.5	10.8	*0.00*	2.2	0.02	-4.4	*0.00*	461	1	1.70	680	*0.14*
15	223	12.3	2750	58.2	6.8	*0.00*	3.2	*0.00*	1.6	0.12	475	1	1.90	223	*0.21*
16	489	7.3	3568	30.3	5.3	*0.00*	1.2	*0.23*	-1.0	0.33	424	2	1.98	282	0.01
17	246	12.1	2967	29.5	10.4	*0.00*	3.5	*0.00*	-1.8	0.08	184	1	1.67	246	0.03
18	415	10.6	4393	30.6	10.6	*0.00*	2.3	*0.02*	-6.8	*0.00*	311	1	1.71	415	*0.21*
19	409	10.8	4416	53.1	8.3	*0.00*	1.4	0.15	-2.2	*0.03*	464	1	1.93	409	0.04
20	381	6.6	2505	34.3	6.3	*0.00*	0.8	0.42	1.0	0.33	396	2	2.19	174	0.03
21	503	10.7	5359	37.2	8.3	*0.00*	3.2	*0.00*	-1.1	0.27	332	2	1.80	281	*0.11*
22	290	13.5	3928	56.1	9.5	*0.00*	1.8	0.07	-1.4	0.18	501	1	1.85	290	0.01
23	629	12.5	7861	52.3	10.4	*0.00*	1.4	0.16	-2.3	*0.02*	501	2	1.87	377	*0.20*
mean	586	10.8	6088		10.0	*0.05>*	1.7	*0.05>*	-3.3	*0.05>*	418	1.4	1.83	451	*0.1<*
s.d.	221	2.5	1933		2.3	*23*	0.8	*8*	3.0	*15*	121	0.5	0.1	169	*7*

n, number of all of the rest bouts; <x>, average length of the rest bout (min); total, total length of rest bout (min); sd, standard deviation of the length of the rest bout (min); LR, log likelihood ratio of the power-law distribution to exponential distribution; p, p-value, positive values of the LR with p<0.05 indicate that the power-law distribution is statistically favored over the alternative distribution. p-values less than 0.05 are indicated in italic. In the power law test, we calculated the p′-value for the best power-law fit for the empirical data set. p′-values larger than 0.1 are indicated in italic. xmax, maximum value of fitted portion; xmin, minimum value of fitted portion; a, exponent of the fitted power law function; ntail, number of bouts with x ≥ xmin.

**Table 2:** Statistical tests for the significance of the power-law property in distributions of rest bouts according to the method described by Clauset et al. (CCFS)

**No.**	**Basic parameters**				**Power Law vs. exponential**						**Power law test**				
					**24h**		**UP**		**DOWN**						
	**n**	**<x>**	**Total**	**sd**	**LR**	**p**	**LR**	**p**	**LR**	**p**	**xmax**	**xmin**	**α**	**ntail**	**p’**
1	2286	7.7	17608	21.8	8.7	*0.00*	2.0	*0.04*	0.7	0.50	301	4	1.91	758	0.02
2	783	7.9	6218	28.7	12.1	*0.00*	0.8	0.41	-0.2	0.83	344	1	1.87	783	0.00
3	449	21.4	9595	56.2	17.4	*0.00*	2.0	*0.04*	-0.8	0.41	426	1	1.61	449	0.00
4	366	12.3	4514	37.1	8.0	*0.00*	1.4	0.15	0.6	0.56	401	1	1.64	366	*0.42*
5	642	8.5	5466	40.4	6.6	*0.00*	1.8	0.07	-1.2	0.23	549	1	1.81	642	0.00
6	509	13.1	6668	53.2	10.8	*0.00*	1.6	0.11	-1.0	0.34	512	1	1.78	509	0.01
7	322	17.5	5646	62.7	6.6	*0.00*	1.4	0.16	-0.3	0.78	592	2	1.69	215	*0.26*
8	965	13.0	12566	43.5	12.3	*0.00*	3.3	*0.00*	-1.5	0.13	541	2	1.73	555	0.05
9	1264	10.8	13604	55.1	12.5	*0.00*	3.0	*0.00*	-1.5	0.14	683	1	1.88	1264	0.00
10	1239	9.3	11475	44.5	13.9	*0.00*	3.2	*0.00*	-2.0	*0.05*	501	2	2.02	631	0.00
11	507	11.2	5678	55.8	5.7	*0.00*	-0.3	0.73	-0.3	0.73	558	2	1.85	298	0.00
12	577	13.5	7806	46.7	15.1	*0.00*	1.6	0.12	-1.7	0.09	371	1	1.76	577	0.02
13	1625	8.9	14494	31.2	16.3	*0.00*	0.1	0.95	-0.6	0.52	356	1	1.79	1625	0.00
14	1168	11.8	13771	40.5	7.6	*0.00*	0.2	0.81	-0.7	0.51	573	7	2.13	383	0.02
15	1050	12.0	12567	49.0	15.4	*0.00*	1.4	0.15	-1.8	0.07	469	1	1.81	1050	0.00
16	1529	10.6	16184	36.0	9.8	*0.00*	4.0	*0.00*	-1.3	0.19	537	3	1.83	696	*0.28*
17	1226	9.9	12123	51.7	11.3	*0.00*	1.1	0.27	-1.5	0.14	592	2	2.00	656	0.00
18	491	9.6	4723	42.9	7.6	*0.00*	2.6	*0.01*	0.4	0.72	470	1	1.83	491	0.00
19	842	10.5	8850	39.0	10.1	*0.00*	1.8	0.07	-0.9	0.38	488	2	1.82	483	*0.73*
20	1787	8.5	15131	36.1	14.9	*0.00*	4.3	*0.00*	-1.4	0.15	479	1	1.87	1787	0.00
21	171	21.9	3741	72.4	9.2	*0.00*	1.0	0.30	0.2	0.85	579	1	1.66	171	0.00
22	572	12.7	7241	40.6	13.0	*0.00*	2.4	*0.02*	-0.2	0.88	375	1	1.70	572	0.02
23	555	10.6	5870	36.3	10.6	*0.00*	1.7	0.09	0.5	0.61	468	1	1.74	555	0.01
24	2244	8.1	18070	33.3	12.3	*0.00*	4.4	*0.00*	-2.3	*0.02*	457	2	1.92	1262	0.00
25	1029	7.5	7738	27.1	9.1	*0.00*	1.2	0.24	-0.7	0.47	351	2	1.92	575	*0.38*
26	1073	11.8	12610	28.8	17.9	*0.00*	1.4	0.17	-1.7	0.09	233	1	1.63	1073	0.00
27	550	10.8	5964	43.5	10.4	*0.00*	4.1	*8.00*	-0.6	0.58	454	2	1.88	294	0.01
28	706	9.2	6495	24.4	10.0	*0.00*	1.7	0.08	-1.3	0.20	227	2	1.80	438	*0.10*
29	545	13.8	7527	32.3	12.3	*0.00*	5.7	*0.00*	0.2	0.82	384	1	1.59	545	0.00
30	1050	9.3	9756	33.4	8.5	*0.00*	4.7	*0.00*	-0.6	0.56	383	3	1.90	463	*0.37*
31	1278	8.9	11314	19.1	3.2	*0.00*	1.3	0.21	-1.2	0.22	207	7	1.99	348	0.00
32	903	10.7	9647	39.3	12.4	*0.00*	2.1	*0.04*	-1.6	0.11	470	1	1.75	903	0.03
33	1994	7.7	15294	35.8	11.0	*0.00*	5.0	*0.00*	-2.5	*0.01*	504	2	1.97	1069	0.00
34	1472	15.7	23068	55.8	5.6	*0.00*	2.7	*0.01*	-1.0	0.31	1007	7	1.83	454	0.01
35	1119	10.8	12141	58.5	11.3	*0.00*	3.1	*0.00*	-1.3	0.18	866	2	2.00	610	0.00
36	543	15.4	8387	58.5	12.8	*0.00*	2.8	*0.01*	-1.6	0.11	501	1	1.73	543	0.00
37	594	11.8	7038	46.9	9.4	*0.00*	2.1	*0.04*	0.2	0.82	568	1	1.73	594	*0.29*
38	1077	10.8	11591	38.0	14.3	*0.00*	2.7	*0.01*	-1.2	0.22	422	1	1.75	1077	0.07
39	1812	10.0	18073	32.8	17.6	*0.00*	2.0	*0.05*	-2.1	*0.04*	345	2	1.84	1051	0.00
40	1690	10.0	16965	27.9	13.9	*0.00*	3.1	*0.00*	-0.5	0.61	372	2	1.75	1009	0.07
41	756	9.2	6991	33.3	8.4	*0.00*	2.6	*0.01*	-0.9	0.35	434	3	1.94	324	0.07
42	1646	10.8	17749	35.0	14.4	*0.00*	1.8	0.07	-1.2	0.24	483	2	1.78	1004	0.09
43	1542	12.0	18481	52.4	13.0	*0.00*	1.4	0.17	-0.8	0.43	809	2	1.82	867	0.00
44	1850	8.2	15235	27.3	12.2	*0.00*	2.2	*0.03*	0.2	0.81	444	3	1.91	739	0.00
45	1542	12.0	18481	52.4	13.0	*0.00*	1.4	0.17	-0.8	0.43	809	2	1.82	867	0.00
46	1096	8.0	8746	31.3	13.5	*0.00*	1.5	0.14	-1.9	0.06	364	2	1.98	568	0.00
47	1316	11.7	15350	49.4	17.5	0.00	2.4	*0.02*	-1.2	0.21	567	2	1.92	727	0.00
48	425	9.8	4153	30.7	7.9	*0.00*	3.8	*0.00*	-0.4	0.70	281	1	1.70	425	*0.18*
49	1159	9.3	10780	26.3	6.8	*0.00*	2.8	*0.01*	-0.8	0.41	267	5	1.90	356	*0.20*
50	785	11.7	9212	39.8	12.1	*0.00*	4.8	*0.00*	-0.2	0.81	439	1	1.70	785	*0.18*
51	580	10.8	6272	58.0	6.8	*0.00*	4.5	*0.00*	-0.9	0.39	704	2	1.94	328	0.02
52	2239	9.4	20994	52.5	10.4	*0.00*	0.9	0.36	-1.6	0.12	720	3	2.06	945	0.00
53	562	16.7	9385	56.3	16.9	*0.00*	1.2	0.22	-0.5	0.63	520	1	1.74	562	0.00
54	916	16.1	14710	50.6	24.4	*0.00*	8.6	*0.00*	-2.3	*0.02*	410	1	1.73	916	0.00
55	798	13.0	10413	37.6	14.2	*0.00*	5.4	*0.00*	0.2	0.85	502	1	1.64	798	0.09
56	1272	8.9	11302	38.0	12.2	*0.00*	2.2	*0.03*	-2.2	*0.03*	396	1	1.83	1272	0.00
57	612	9.8	5996	39.4	5.6	*0.00*	0.8	0.45	0.9	0.39	435	4	2.05	250	0.01
mean	1047	11.3	11008		11.5	*0.05>*	2.5	*0.05>*	-0.9	*0.05>*	482	2.0	1.83	694	*0.1<*
s.d.	530	3.1	4761		3.9	*57*	1.6	*31*	0.8	*6*	156	1.5	0.1	341	*11*

n, number of all of the rest bouts; <x>, average length of the rest bout (min); total, total length of rest bout (min); sd, standard deviation of the length of the rest bout (min); LR, log likelihood ratio of the power-law distribution to exponential distribution; p, p-value, positive values of the LR with p<0.05 indicate that the power-law distribution is statistically favored over the alternative distribution. p-values less than 0.05 are indicated in italic. In the power law test, we calculated the p′-value for the best power-law fit for the empirical data set. p′-values larger than 0.1 are indicated in italic. xmax, maximum value of fitted portion; xmin, minimum value of fitted portion; a, exponent of the fitted power law function; ntail, number of bouts with x ≥ xmin.

Thus, we submitted the 24 h rest data to a strict power law test developed by Clauset et al. [10]. As described in the materials and methods, we fitted the individual data to a power law distribution and generated 1,000 surrogate data sets, each of which was a set of artificial bouts sampled from the estimated power-law distribution with noise. Then we compared these surrogate data with the actual data and calculated a *p′-*value for the best power-law fit, which was used for the judgment. Note that we deemed the distribution to be significantly similar to the power law when this *p′-*value was larger than 0.1. As shown in Table 1a and b, the power law distribution that was fitted to the actual data spans from 1 to 2 min (xmin) to about 400 min (xmax) and the α in Figure 4c, approximately 30% of control and 20% of CCFS subjects showed a *p′* value larger than 0.1. These proportions were significantly smaller when compared with the *Drosophila* study, where all the individuals showed significant similarity to the power law distributions [9].

## Discussion

We analyzed the temporal organization of rest and activity bouts using actigraphy data from both healthy youngsters and individuals with CCFS. We applied a k-means clustering method to calculate the threshold values and compared them with those calculated by the average method used by Nakamura et al. As shown in Figure 1a, the k-means clustering method gave smaller values than the average method, but with significant correlations. Nakamura et al. reported that changing the threshold value within the range of 0.6 to 1.6 fold did not affect the results [3]. However, when we analyzed our data using the threshold value, which corresponded to 1.6 fold of the value calculated by the average method, most of the bins were judged as ‘rest’ even during daytime (UP period), thus, we could not obtain satisfactory results (data not shown). Since we did not directly compare the actual data, we do not know why this occurred. However, we speculate that differences in the sensitivity or properties of the actigraphy could be the reason. The threshold values calculated by the k-means clustering method were smaller, and stable among the subjects as shown in Figure 2a. Therefore, we decided that the k-means clustering method was more appropriate for our data so we chose to use it in this study. We anticipated no essential differences in the results, depending on the calculation method used for the threshold.

The distribution of rest over 24 h showed long-tailed distributions and when the power law and exponential distributions were compared, all the individual data significantly favored the power law (Figure 4a, Tables 1, 2). These results are consistent with the previous analysis from Nakamura et al. [3]. However, the distribution curve showed a flexion (Figures 1c and 3a,e,i) which was not evident when we analyzed the fruit fly data. Since we expected longer rest to be derived from nighttime sleep, we re-analyzed the data after separation into UP and DOWN periods. The results showed that the rest bout during UP periods had a weak similarity to the power law distribution, but the rest bout during DOWN periods did not (Figure 4a, Table 1). Even for the rest bout of 24 h, which favored the power law over the exponential distribution, only 30% passed the strict power law judgment proposed by Clauset et al. [10]. One of the most famous examples of power law properties in animal behavior was Levy flight described by Viswanathan [19]. However, the amount of data presented in that paper was limited, therefore the power law properties reported have been questioned [20]. In addition, recently, Petrovskii et al. reported that the summations of individual data, which alone show exponential and non-power law distributions, can reveal power law properties [21]. Thus, we should pay more attention to the interpretation of power law properties for 24 h rest bouts.

The rest bout during DOWN periods, which corresponds to nighttime sleep, tended to show a similarity to an exponential distribution in healthy control subjects (Figure 4c). This is consistent with the fact that human sleep consists of repeating sleep units. It is also consistent with a previous report by Lo et al., which used an electroencephalogram to demonstrate a sleep bout that showed an exponential distribution [12].

As we described previously, in fruit fly, rest period showed a clear power law distribution even when the daytime and nighttime data were analyzed separately [9]. The difference in the temporal distribution of rest between human and fruit fly may reflect the qualitative difference of rest and sleep between them.

From the clinical point of view, actigraphy has been broadly used as a versatile tool to monitor activity and sleep rhythm in normal and patients with psychiatric and other disorders [13-16]. The temporal distribution of activity and rest were described to show difference between normal and patients with disorders. Recently, Sano et al described enhanced persistence of rest and active periods in schizophrenia patients [22].

Analysis of CCFS children did not disclose difference from normal subject when analyzed as a whole day data. But, the rest bout during DOWN periods in CCFS subjects showed a reduced tendency (60% in the control, 10% in CCFS) to favor the exponential distribution (Figure 4c). Since the average plots for these two groups almost overlap (Figure 3i-l), it is interesting to find such a large difference in the individual analysis. This may be due to the instability of sleep in CCFS children and could be used as pathological marker for this condition. However, more studies are necessary to find the exact cause and clinical value.

In conclusion, although power law properties in animal behavior have attracted attention since they are apparently universal, we should pay more careful attention when interpreting power law properties of the data, since apparent power law property can be derived from the summation of two non-power law distributions.

## Conclusion

We analyzed the temporal organization of rest and activity bouts using actigraphy data from both healthy youngsters and individuals with CCFS, after separating them into UP and DOWN periods. The rest durations of either UP or DOWN period did not show a power law distribution. We propose that separate analysis of UP and DOWN period data is important for understanding the temporal organization of activity.

## Competing interests

The authors declare they have no competing interests.

## Authors’ contributions

All authors contributed to and approved the final version of the manuscript. MK, TU, JT, JK, AT, SK and KK designed the study. JK and AT acquired the data, MK, TU, JT, SK and KK conducted the data analysis, interpretation and drafted the manuscript. All authors read and approved the final manuscript.

## Authors’ information

TU, JK, AT and KK are medical doctors, and TU and KK are physicians and JK and AT are pediatricians. MK and SK are pharmacists. KK is a certified physician of sleep medicine accredited by the Japanese Society of Sleep Research (JSSR). As of 2012, there are 413 accredited in Japan.

## Pre-publication history

The pre-publication history for this paper can be accessed here:

http://www.biomedcentral.com/1471-244X/13/281/prepub
